# Sialic acid-binding immunoglobulin-like lectin-15 expression on peritumoral macrophages is a favorable prognostic factor for primary central nervous system lymphoma patients

**DOI:** 10.1038/s41598-020-79742-9

**Published:** 2021-01-13

**Authors:** Hirotaka Fudaba, Yasutomo Momii, Taisei Hirakawa, Kouhei Onishi, Daigo Asou, Wataru Matsushita, Yukari Kawasaki, Kenji Sugita, Minoru Fujiki

**Affiliations:** 1 Department of Neurosurgery, Oita University Faculty of Medicine, 1-1 Idaigaoka, Hasamamachi, Yufu, 879-5593 Japan; 2 School of Medicine, Oita University Faculty of Medicine, Yufu, 879-5593 Japan

**Keywords:** Cancer microenvironment, CNS cancer

## Abstract

Sialic acid-binding immunoglobulin-like lectin-15 (Siglec-15) is a new immune checkpoint molecule and its role of primary central nervous system lymphoma (PCNSL) tumor microenvironment has been unclear. We explored the Siglec-15 and programed death-ligand 1 (PD-L1) expression in tumor tissues and analyzed the association between the expression of these molecules and overall survival in newly diagnosed PCNSL. A total of 60 patients diagnosed with diffuse large B-cell lymphoma in PCNSL were included in this study. The Siglec-15 and PD-L1 expression on tumor cells, intratumoral macrophages and peritumoral macrophages were immunohistochemically evaluated. The expression of Siglec-15 and PD-L1 was greater in macrophages than in tumor cells. Regarding peritumoral macrophages, the number of Siglec-15-positive samples (n = 24) was greater than the number of PD-L1-positive samples (n = 16). A multivariate Cox analysis showed that the Siglec-15 positivity of peritumoral macrophages and performance of high-dose methotrexate-based chemotherapy were independent predictors of overall survival (hazard ratio: 0.295 and 0.322, respectively). The Kaplan–Meier survival curves showed that patients with Siglec-15-positive peritumoral macrophages had longer overall survival than those with Siglec-15-negative peritumoral macrophages (median overall survival: 3018 days and 746 days, respectively; *p* = 0.0290). Our findings indicate that the expression of Siglec-15 on peritumoral macrophages induces a favorable outcome in PCNSL patients.

## Introduction

The histopathological diagnosis of primary central nervous system lymphoma (PCNSL) is mostly limited to diffuse large B-cell lymphoma (DLBCL). DLBCL in the central nervous system (CNS) accounts for 2.4–3% of all brain tumors and their prognosis is poor, even with the administration of multimodal therapy^1^. The standard therapy for DLBCL in the CNS is high-dose methotrexate chemotherapy followed by whole brain radiotherapy, although high-dose methotrexate-based polychemotherapy, such as the R-MPV regimen (rituximab, methotrexate, procarbazine, and vincristine), with reduced whole brain radiation therapy, improves their prognosis^2^. However, based on a recent report, whole brain radiotherapy seems to be contraindicated in elderly patients due to their declining cognitive function^3^.

Immune checkpoint molecules are targetable in the treatment of malignant and refractory disease. The programed death-1 (PD-1)/the programed death-ligand 1 (PD-L1) pathway is a well-known target of new treatments and other immune checkpoint molecules have been reported, including—but not limited to—cytotoxic T lymphocyte-associated antigen-4 (CTLA-4), lymphocyte activation gene-3 (LAG-3), T cell immunoglobulin and mucin-domain containing-3 (Tim-3)^4^. A small retrospective study reported that a long-term response was achieved in PCNSL patients treated with anti-PD-1 monoclonal antibodies^5^. A single institution trial of pembrolizumab (NCT02779101) to further investigate the concept of immune evasion and PD-1 blockage in PCNSL is currently ongoing.

In addition, a recent publication reports a new immune suppressor, sialic acid-binding immunoglobulin-like lectin-15 (Siglec-15)^6^. Siglec-15 is a member of the Siglec family and is expressed in a wide variety of tumor cells and tumor-associated macrophages. Siglec-15 has similar immunomodulatory functions to PD-L1; however, the expression of Siglec-15 and PD-L1 is mutually exclusive, suggesting that Siglec-15 antibodies may be effective for tumors that are not responsive to anti-PD-1/PD-L1 therapy. Siglec-15 has been reported to activate the AKT pathway through DAP12^7^. It has previously been demonstrated that Siglec-15 recognizes the tumoral the sialyl-Tn antigen and transduces a signal for enhanced transforming growth factor-β (TGF-β) secretion in tumor-associated macrophages and suggests that the expression of Siglec-15 on macrophages may contribute to tumor progression by the TGF-β-mediated modulation of the intratumoral microenvironment^8^. A clinical trial is currently ongoing to test the efficacy of NC318 (an anti-Siglec-15 monoclonal antibody) in solid tumors (NCT03665285).

Other investigations have shown that immune checkpoint molecules have applications as prognostic factors; however, the findings of the reports remain unclear and paradoxical. A meta-analysis revealed the PD-L1 expression on tumor cells was associated with a worse prognosis in solid tumors^9^. However, another article demonstrated that PCNSL patients with PD-L1-positive tumor cells had a better prognosis than those with PD-L1-negative tumor cells^10^. Recently, the immune checkpoint molecules expressed on tumor infiltrating immune cells, such as T cells, macrophages and dendritic cells have received attention. In head and neck cancer patients, the expression of PD-L1 on tumor infiltrating immune cells, but not on tumor cells is an independent predictor of favorable overall survival^11^. Another meta-analysis evaluated the prognostic value of the PD-L1 expression in tumor infiltrating immune cells and suggested that PD-L1 positivity in tumor infiltrating immune cells indicated a better prognosis in breast cancer patients^12^. With regard to lymphoma, recent studies have shown that the PD-L1 expression on peritumoral macrophages was strongly predictive of a favorable outcome^13^. However, there are no reports analyzing the association between the expression of Siglec-15—which seems to be mutually exclusive against PD-L1—on tumor cells and macrophages with the survival in patients with PCNSL.

Thus, in the present study, we investigated the Siglec-15 and PD-L1 expression on tumor cells, intratumoral macrophages and peritumoral macrophages using immunohistochemistry. Our purpose was to evaluate whether or not the expression of these immune checkpoint molecules in the tumor microenvironment provided useful, complementary information, and whether or not these parameters showed the best performance in predicting the outcomes of newly diagnosed PCNSL.

## Results

The 60 patients included 27 males and 33 females with a mean age of 69.5 ± 9.2 years. The mean follow-up period was 897 days (range 69–4306 days) and the median Karnofsky performance status (KPS) was 70 (range 40–90). The peritumoral tissue was involved in 55 tissue samples and not involved in 5 samples. The patient characteristics and treatment characteristics of this study cohort are shown in Table 1.

**Table 1:** Patient characteristics.

	Total (n = 60) n (%)
**Age (years)**	
< 70	29 (48)
≥ 70	31 (52)
**Sex**	
Male	27 (45)
Female	33 (55)
**Molecular subgroup**	
Non-GCB type	33 (55)
GCB type	16 (27)
NA	11 (18)
**MSKCC score**	
Class 1	0 (0)
Class 2	40 (67)
Class 3	20 (33)
**Extent of resection**	
Partial removal	28 (47)
Biopsy	32 (53)
**Induction chemotherapy**	
Rituximab	43 (72)
High-dose methotrexate	12 (20)
R- MPV	18 (30)
R- CHOP and CHOP	21 (35)
R- THP- COP and THP- COP	6 (10)
Radiation at induction therapy	27 (45)

CHOP, cyclophosphamide, hydroxydaunorubicin, vincristine, and prednisolone; GCB, germinal center B-cell; MPV, methotrexate, procarbazine, and vincristine; MSKCC, Memorial Sloan Kettering Cancer Center; R, rituximab; THP- COP, cyclophosphamide, pirarubicon, vincristine, and prednisolone.

### The expression of Siglec-15 and PD-L1 on tumor cells and macrophages in the tumor tissue

**Figure 1:**
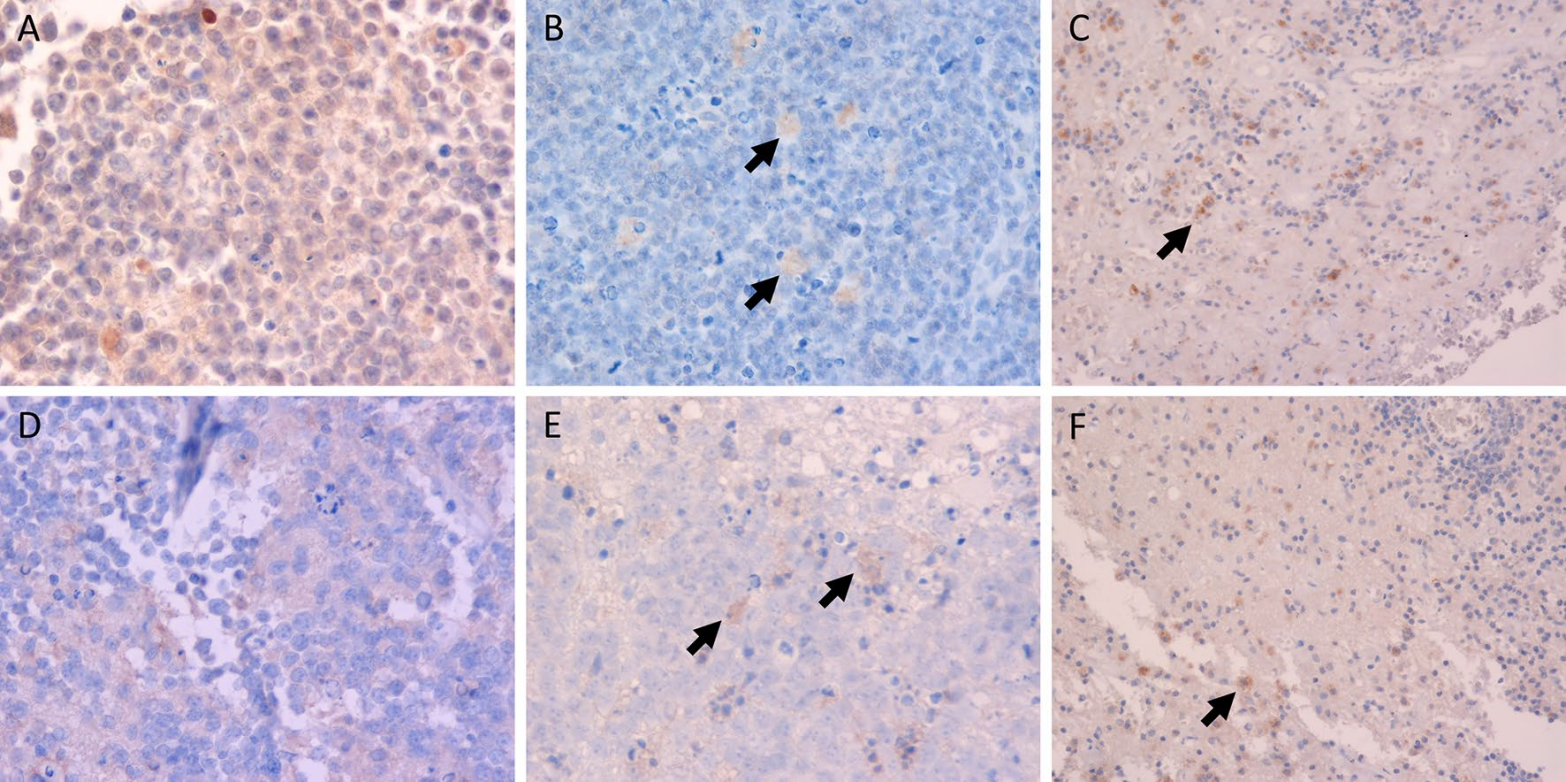
The expression of Siglec-15 (**A** positive tumor cells, **B** positive intratumoral macrophages, **C** positive peritumoral macrophages) and the expression of PD-L1 (**D** positive tumor cells, **E** positive intratumoral macrophages, **F** positive peritumoral macrophages) in tumor tissue (**A**,**B**,**D**,**E** magnification × 400, and **C**,**F** magnification × 200). Dark red-brown color represented the presence of antigens and antigen-positive cells indicated with arrows in the figures.

**Figure 2:**
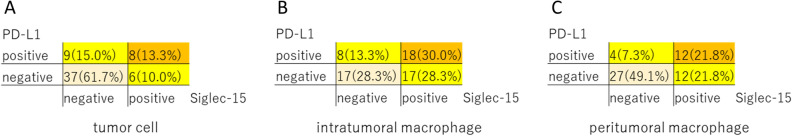
The correlation of the Siglec-15 and PD-L1 expression in our cohort (**A** tumor cells, **B** intratumoral macrophages, **C** peritumoral macrophages). In the majority of the patients (37 samples) tumor cells were negative for Siglec-15 and PD-L1. Larger numbers of samples had Siglec-15- and PD-L1-positive macrophages in comparison to tumor cells. With regard to the expression on peritumoral macrophages, the number of Siglec-15-positive samples (n = 24) was greater than the number PD-L1-positive samples (n = 16).

**Figure 3:**
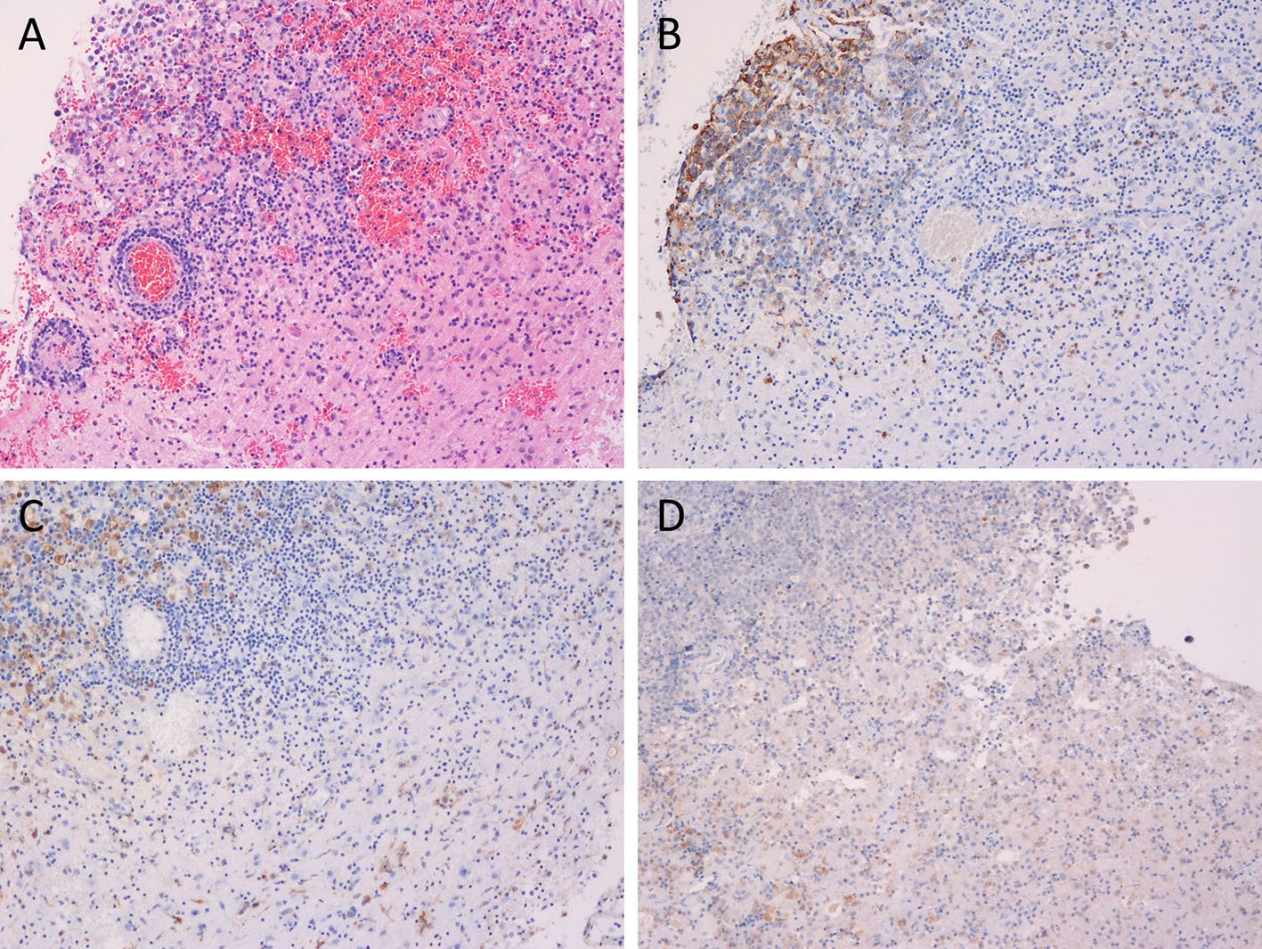
The histopathological findings in the tumoral and peritumoral tissue. Hematoxylin and eosin staining showed the high cellularity, diffuse growth, and perivascular spread of the tumor cells (**A**). The tumor cells were positive for CD20 (**B**). CD68 staining revealed the existence of intratumoral macrophages and peritumoral macrophages (**C**). The macrophages were positive for Siglec-15 while the tumor cells were negative for Siglec-15 (**D**). (magnification × 100). Dark red-brown color represented the presence of antigens in (**B**–**D**).

**Figure 4:**
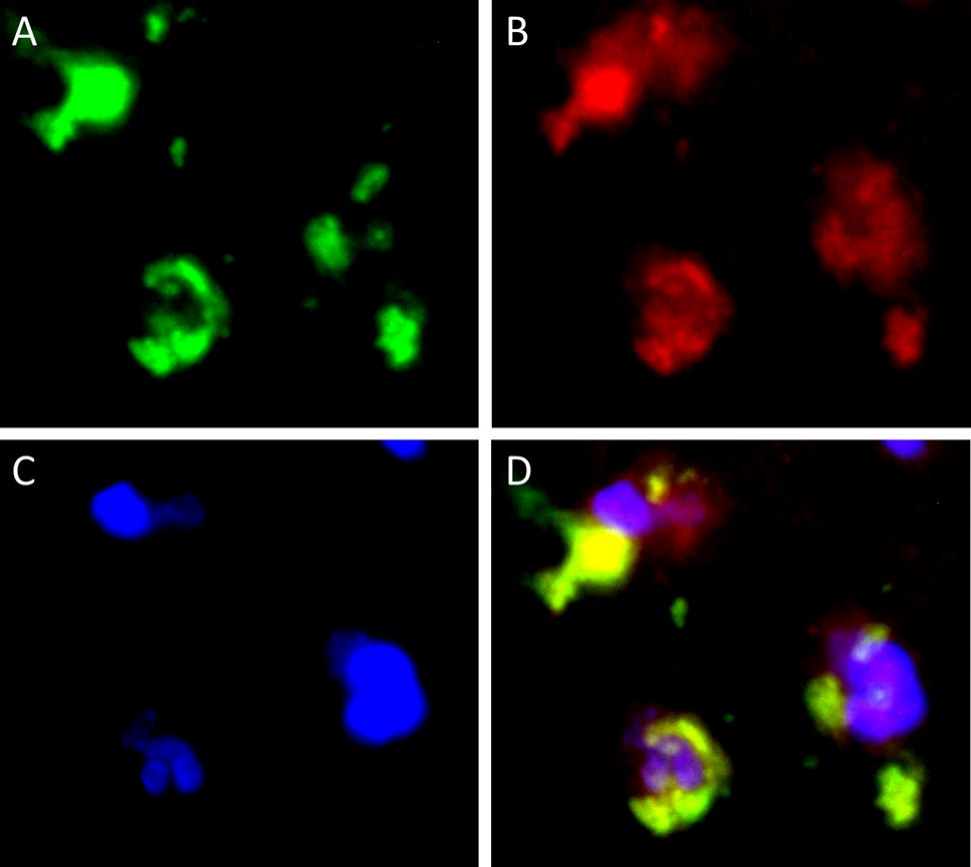
Double-immunofluorescent staining of CD68 and Siglec-15. CD68 staining (**A** green). Siglec-15 staining (**B** red). DAPI staining (**C** blue). Merged images (**D**). Representative images of PCNSL peritumoral tissue with the co-expression of CD68 and Siglec-15. (magnification × 1000).

Among the 60 tumor samples, 14 samples showed Siglec-15 positivity and 17 showed PD-L1 positivity in tumor cells (Fig. 1a,d. Figure 2a). In the majority of patients (37 samples), the samples were negative for Siglec-15 and PD-L1; only 8 samples were positive for Siglec-15 and PD-L1. Regarding macrophages in tumor tissue, 35 samples had Siglec-15-positive intratumoral macrophages and 24 samples had Siglec-15-positive peritumoral macrophages (Figs. 1b,c, 2b,c). Twenty-six samples had PD-L1-positive intratumoral macrophages and 16 samples had PD-L1-positive peritumoral macrophages (Figs. 1e,f, 2b,c). Intratumoral macrophages were positive for both Siglec-15 and PD-L1 in 18 tumor tissue samples and negative for both Siglec-15 and PD-L1 in 17 tumor tissue samples (Fig. 2b). Peritumoral macrophages were positive for both Siglec-15 and PD-L1 in 12 tumor tissue samples, and negative for both Siglec-15 and PD-L1 in 27 tumor tissue samples (Fig. 2c). Images of Siglec-15 antibody staining of human tonsil and temporal lobe tissue specimens, which were used as positive and negative controls (see Supplementary Fig. S1 online). A representative case is shown in Fig. 3. Double-immunofluorescent staining for Siglec-15 and CD68 was observed in peritumoral macrophages (Fig. 4).

### Association between the Siglec-15 and PD-L1 expression and overall survival

**Figure 5:**
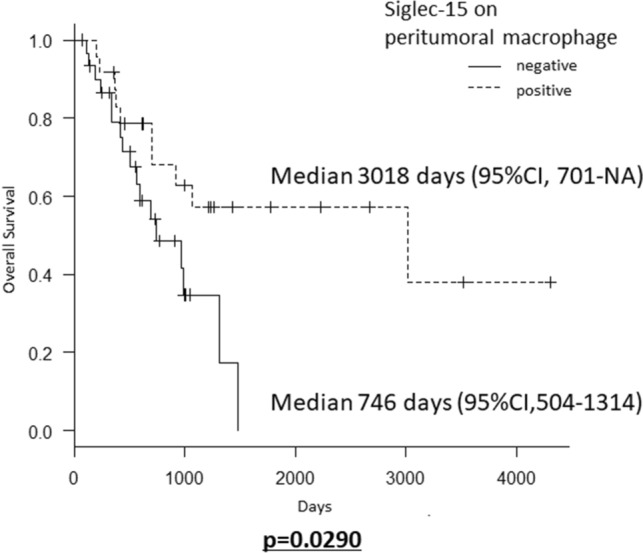
Kaplan–Meier survival curves for PCNSL patients stratified by whether or not peritumoral macrophages expressed Siglec-15. *CI* confidence interval, *NA* not analyzed.

Regarding the Siglec-15 expression, the median overall survival was significantly longer in cases with Siglec-15-positive peritumoral macrophages (median survival time, 3018 days; 95% CI, 701 days-NA) in comparison to the patients with Siglec-15-negative peritumoral macrophages (median survival time, 746 days; 95% CI, 504–1314 days) (*p* = 0.0290, Fig. 5). However, the positive and negative expression of Siglec-15 on tumor cells and intratumoral macrophages had no significant effect on overall survival.

Regarding the expression of PD-L1, the median overall survival was significantly longer in the PD-L1-positive tumor cell group (median survival time, 3018 days; 95% CI, 993 days-NA) in comparison to the PD-L1-negative group (median survival time, 746 days; 95% CI, 565–1314 days) (*p* = 0.0289). In addition, the overall survival of the PD-L1-positive and PD-L1-negative intratumoral macrophage groups and the PD-L1-positive and PD-L1-negative peritumoral macrophage groups did not differ to a statistically significant extent.

The results of univariate Cox regression analyses that included biological and treatment factors are shown in Table 2. The univariate analysis identified the following significant factors: the positive expression of Siglec-15 on peritumoral macrophages (HR = 0.404, 95%CI: 0.175–0.933, *p* = 0.0339), the positive expression of PD-L1 on tumor cells (HR = 0.373, 95%CI: 0.149–0.931, *p* = 0.0347) and performance of high-dose methotrexate-based chemotherapy (HR = 0.417, 95%CI: 0.183–0.950, *p* = 0.0373). No significant correlations were noted between other parameters (Table 2).

**Table 2:** Results of the univariate Cox survival analyses.

Factor	Hazard ratio (95%CI)	*p* value
Age ≥ 70	1.636 (0.782–3.426)	0.192
Molecular subgroup (GCB type vs Non-GCB type)	0.891 (0.359–2.213)	0.804
MSKCC score (class 3 vs class 1 and 2)	1.049 (0.479–2.295)	0.905
Siglec-15 positive tumor cells	1.241 (0.570–2.699)	0.586
Siglec-15 positive intratumoral macrophages	1.713 (0.783–3.748)	0.178
Siglec-15 positive peritumoral macrophages	0.404 (0.175–0.933)	0.0339
PD-L1 positive tumor cells	0.373 (0.149–0.931)	0.0347
PD-L1 positive intratumoral macrophages	1.369 (0.666–2.817)	0.393
PD-L1 positive peritumoral macrophages	0.585 (0.245–1.398)	0.227
Rituximab	0.999 (0.464–2.154)	0.999
High-dose methotrexate-based chemotherapy	0.417 (0.183–0.950)	0.0373

CI, confidence interval; GCB, germinal center B-cell; MSKCC, Memorial Sloan Kettering Cancer Center.

Multivariate Cox regression analyses revealed that the Siglec-15 positive expression on peritumoral macrophages (HR = 0.295, 95%CI: 0.125–0.697, *p* = 0.0054) and the performance of high-dose methotrexate-based chemotherapy (HR = 0.332, 95%CI: 0.135–0.770, *p* = 0.0108) independently predicted survival in patients with DLBCL (Table 3).

**Table 3:** The results of multivariate Cox regression analyses.

Factor	Hazard ratio (95%CI)	*p* value
Siglec-15 positive peritumoral macrophages	0.295 (0.125–0.697)	0.0054
Performance of high-dose methotrexate-based chemotherapy	0.322 (0.135–0.770)	0.0108

CI, confidence interval.

We obtained the Siglec-15 mRNA data through UALCAN based on the TCGA database. The expression of Siglec-15 mRNA in DLBCL tended to be higher than that in other types of tumors (see Supplementary Fig. S2 online). A log-rank test that included 47 patients with DLBCL, in cases in which samples were obtained from whole body, revealed that the group with the higher expression of Siglec-15 mRNA had longer overall survival than the other group; however, this difference was not statistically significant (*p* = 0.088) (see Supplementary Fig. S3 online).

## Discussion

PCNSL is a highly aggressive tumor and has a favorable response to both chemotherapy and radiation therapy. The first-line therapy for DLBCL occurring outside the CNS is R-CHOP (cyclophosphamide, doxorubicin, vincristine, and prednisolone); however, DLBCL in the CNS is treated with the introduction of high-dose methotrexate chemotherapy in combination with whole brain radiation therapy. High-dose methotrexate-based polychemotherapy, such as the R-MPV regimen followed by reduced whole brain radiotherapy has recently been reported to show a favorable outcome^2^. For patients with recurrent and refractory PCNSL, Bruton tyrosine kinase (BTK) inhibitor and immunomodulatory drugs, such as lenalidomide and pomalidomide, have produced more promising results^14^. Another promising approach might be the use of immune checkpoint inhibitors. A single institution trial with pembrolizumab is ongoing to investigate the concept of PD-1 blockade in PCNSL (NCT02779101). Our study, which focused on PD-L1 and Siglec-15, suggests that Siglec-15 is also targetable molecule for the regulation of the tumor microenvironment in patients with PCNSL.

Various studies have investigated the PD-L1 expression in the PCNSL microenvironment. According to past reports, the expression of PD-L1 is more frequently detected on tumor infiltrating cells, including macrophages than tumor cells^10,13,15–17^. Our results show similar trends. Regarding the association between PD-L1 and survival in patients with PCNSL, the past literature showed that the PD-L1 expression in tumor cells was correlated with overall survival and that the median survival time was not reached among patients with PD-L1-positive tumor cells and 31.7 months among those with PD-L1-negative tumor cells, although the PD-L1 expression in tumor stromal cells was not correlated with overall survival^10^. On the other hand, Furuse et al. analyzed the expression of PD-L1 and PD-L2 in both tumor and peritumoral tissues and revealed that PD-L1-positivity of intratumoral and peritumoral macrophages predicted a favorable outcome^13^. Our results showed that tumor cell PD-L1 positivity was associated with longer overall survival in comparison to PD-L1 negativity; however, this factor was excluded by a multivariate Cox regression analysis.

Siglec-15 has been shown to be involved in osteoclast differentiation, and is considered to be a potential therapeutic target for osteoporosis^7,18^. Recent studies revealed the immunosuppressive role of Siglec-15 and identified Siglec-15 as a potential target for normalization cancer immunotherapy^6^. Siglec-15 is expressed on tumor cells and tumor-associated macrophages and plays a role as ligand for an unknown inhibitory receptor on cytotoxic T cells like PD-L1. Interestingly, the expression of Siglec-15 and PD-L1 are mutually exclusive in human lung cancer tissues. It is considered that this is because the induction of Siglec-15 on macrophages is suppressed by interferon-γ while PD-L1 is induced by interferon-γ^6^. In the present study, Siglec-15 was more frequently expressed on macrophages than on tumor cells, which was similar to the expression of PD-L1, although the co-expression of Siglec-15 and PD-L1 was detected on 30.0% of intratumoral macrophages and 21.8% of peritumoral macrophages in PCNSL tissues. These results suggested that the expression of immune checkpoint molecules in the tumor microenvironment varied in different tumor tissues and demonstrates the need for further investigation with the addition of a single-cell analysis.

A recent bioinformatics analysis on various tumor tissues showed that the high expression of Siglec-15 mRNA was associated with worse overall survival in pancreatic ductal adenocarcinoma, sarcoma and kidney renal clear cell carcinoma, while the opposite result is true in breast cancer, head and neck squamous cell carcinoma, thyroid carcinoma, and uterine corpus endometrial carcinoma^19^. With the use of the UALCAN database, the high expression of Siglec-15 mRNA in DLBCL tended to be associated with a favorable outcome. Interestingly, using this database. we could demonstrate that the Siglec-15 mRNA expression of DLBCL was higher in comparison to other tumors. Based on these results Siglec-15 become a therapeutic target in DLBCL. Various reports have discussed the association of the immune checkpoint molecule expression on tumor cells and/or macrophages and overall survival; however, no reports have mentioned the correlation between the Siglec-15 expression on macrophages and overall survival in patients with PCNSL^9–13^. A study of primary testicular lymphoma found that patients with the high expression of PD-L1 on macrophages showed favorable survival^20^. Another study demonstrated the PD-L1 expression on peritumoral macrophages was strongly predictive of a favorable outcome^13^. However, in our study, the expression of Siglec-15, which was reported to be mutually exclusive against PD-L1, on peritumoral macrophages was shown to be a significant prognostic factor, while the PD-L1 expression on macrophages was not. Our present results are consistent with the reports about the association between macrophages and immune checkpoint molecules, which reported that the evaluation of the immune checkpoint molecules expression on macrophages in PCNSL tissues was useful for predicting overall survival. To resolve this discrepancy, is a need for further prospective studies in which immune checkpoint molecules are consistently evaluated and in which a consistent treatment strategy is applied. The reason why immune checkpoint molecules on macrophages are correlated with the tumor prognosis are unclear. A recent study revealed that PD-L1 expressed on macrophages did not inhibit the T cell response, rather, it merely protected macrophages from destruction by T cells, unlike PD-L1 expressed on tumor cells^21^. A previously mentioned study on the regulation of macrophage proliferation and activation by PD-L1 showed that macrophages with high PD-L1 expression levels had greater proliferation, survival, and activation abilities after anti-PD-L1 antibody treatment^22^. These findings suggested the importance of the effect of PD-L1 as a receptor on host macrophages after interaction with its ligand, PD-1, and were helpful for resolving the question as to why patients with the high expression of PD-L1 on macrophages had a better prognosis. Similarly to the function of PD-L1 on macrophages, Siglec-15 may play a role as receptor on host macrophages and induce a favorable outcome in patients with PCNSL.

In conclusion, the findings of the present study indicate that the expression of Siglec-15 on peritumoral macrophages and the performance of high-dose methotrexate-based chemotherapy are associated with favorable outcomes and are useful for predicting the overall survival of patients with DLBCL in the CNS. Further studies are needed to investigate the function of Siglec-15 and tumor-associated macrophages.

## Methods

### Patients

A total of 60 patients (age, 50–84 years) who were newly diagnosed with DLBCL in the primary central nervous system between April 2008 and April 2020 were included in this study. A histological analysis of tissue samples obtained at the time of either surgical resection or image-guided biopsies was performed according to the WHO brain tumor classification (2016 revision)^1^. After the histopathological diagnosis, all patients underwent chemotherapy and/or radiotherapy if permitted by the patient’s condition. If tumor progression or recurrence occurred, salvage chemotherapy and/or additional radiotherapy was considered.

All patients provided their informed consent to participate in this study, which was conducted in accordance with the ethical principles of the Declaration of Helsinki. The study protocol was approved by the Ethics Committee of the Oita University Faculty of Medicine (approval number, 1926).

### Immunohistological assays

For immunostaining, formalin-fixed, paraffin-embedded tissue specimens were sliced into 3-µm-thick sections. We manually stained the sections with a rabbit anti-Siglec-15 polyclonal antibody (PA5-72765; Invitrogen, Carlsbad, CA, USA; 1:500 dilution) and a rabbit anti-PD-L1 monoclonal antibody (ab205921; Abcam, Cambridge, MA, USA; 1:500 dilution) by the standard avidin–biotin complex method. Bound antibodies were visualized using a DAB detection kit (049-22831; Wako). Other antibodies, such as CD3 (LN10; Leica), CD5 (4C7; Leica), CD10 (56C6; Leica), CD20 (L26; Leica), CD68 (514H12; Leica), CD79a (11D10; Leica), BCL6 (LN22; Leica), MUM1 (EAU32; Leica) were stained using an automated staining system (BOND III, Leica Biosystems, Nussloch, Germany). The membranous Siglec-15 and PD-L1 expression on tumor cells and macrophages were evaluated. The positive expression and negative expression were judged by the expression on ≥ 1% or < 1% of tumor cells, respectively. In cases with specimens that included tumor tissue and peritumoral tissue, we evaluated whether Siglec-15 and/or PD-L1 were expressed on peritumoral macrophages. Human tonsil and temporal lobe tissue from the surgical material was used as a positive and negative control, respectively. Immunoreactivity for CD5, CD10, BCL-6 and MUM1 was judged as positive if > 30% of tumor cells were stained. Tumors were subclassified according to the expression pattern of the germinal center B-cell (GCB) and non-GCB markers^23^. The eligibility of immunohistochemically-stained slides was evaluated independently by two authors (H.F. and T.H.), and any disagreements were resolved by consensus.

Fluorescent double immunostaining was performed to detect the Siglec-15 and CD68 expression in tumor tissue. We stained the sections with a rabbit anti-Siglec-15 polyclonal antibody and a mouse anti-CD68 monoclonal antibody (14-0688-82; Invitrogen; 1:500 dilution), followed by the addition of anti-rabbit IgG-Alexa594 (R37117; Invitrogen) and anti-mouse IgG-Alexa488 (R37120; Invitrogen). Nuclei were counterstained with DAPI (P36966; Invitrogen). Fluorescence was analyzed using a fluorescence microscope (BZ-9000, KEYENCE, Osaka, Japan).

### UALCAN database

UALCAN (http://ualcan.path.uab.edu) is a comprehensive, user-friendly, and interactive web resource for analyzing cancer OMICS data^24^. In this study, the UALCAN database was used to obtain data from the TCGA database and to compare the expression of Siglec-15 mRNA in 33 types of cancer, including 47 patients with DLBCL. In addition, we compared the overall survival of 47 DLBCL patients who were included in the database according to whether or not Siglec-15 was expressed.

### Statistical analyses

All statistical analyses were performed using the EZR software program (version 1.40)^25^. Kaplan–Meier survival curves and log-rank tests were used to compare survival until death or the last follow-up examination according to the expression of Siglec-15 and PD-L1 in tumor cells, intratumoral macrophages, and peritumoral macrophages.

Univariate and multivariate survival analyses were performed according to the Cox proportional hazards regression model.* p* values of < 0.05 were considered to indicate statistical significance.

## Supplementary Information

Supplementary Legends.

Supplementary Figure S1.

Supplementary Figure S2.

Supplementary Figure S3.
