# Supplementary material for: Sialic acid-binding immunoglobulin-like lectin-15 expression on peritumoral macrophages is a favorable prognostic factor for primary central nervous system lymphoma patients
Source: Sci Rep. 2021 Jan 13;11:1206. doi: 10.1038/s41598-020-79742-9 (PMC7806611; doi:10.1038/s41598-020-79742-9)
Supplement: Supplementary file 1 — Supplementary Legends. [file 41598_2020_79742_MOESM1_ESM.doc]

**Sialic acid-binding immunoglobulin-like lectin-15 expression on peritumoral macrophages is a favorable prognostic factor for primary central nervous system lymphoma patients**

Hirotaka Fudaba^1*^, Yasutomo Momii^1^, Taisei Hirakawa^2^, Kouhei Onishi^1^, Daigo Asou^1^, Wataru Matsushita^1^, Yukari Kawasaki^1^, Kenji Sugita^1^ and Minoru Fujiki^1^

^1^ Department of Neurosurgery, Oita University Faculty of Medicine, Yufu, 879-5593 Japan

^2^ Department of Medicine, Oita University Faculty of Medicine, Yufu, 879-5593 Japan

**For submission to: Scientific Reports (Original articles)**

Please address correspondence to: HIROTAKA FUDABA MD, PhD.

Department of Neurosurgery, University Faculty of Medicine

Address: 1-1 idaigaoka, hasamamachi

Post code: 879-5593 City: Yufu Country: Japan

Telephone: +81-97-586-5862 Fax: +81-97-586-5869

Email: fudaba@oita-u.ac.jp

Supplementary Figures

Supplementary Figure S1:

The staining with Siglec-15 antibody of human tonsil and temporal lobe tissue (A: positive on human tonsil, B: negative on temporal lobe tissue) (magnification x400).

Supplementary Figure S2:

The expression of Siglec-15 across TCGA tumors. ACC, Adrenocortical carcinoma; BLCA, Bladder urothelial carcinoma; BRCA , Breast invasive carcinoma; CESC, Cervical squamous cell carcinoma; CHOL, Cholangiocarcinoma; COAD, Colon adenocarcinoma; DBLC, Lymphoid Neoplasm Diffuse Large B-cell Lymphoma; ESCA, Esophageal carcinoma; GBM, Glioblastoma multiforme; HNSC, Head and Neck squamous cell carcinoma; KICH, Kidney Choromophobe; KIRC, Kidney renal clear cell carcinoma; KIRP, Kidney renal papillary cell carcinoma; LGG, Brain lower grade glioma; OV, Ovarian serous cystadenocarcinoma; MESO, Mesothelioma; LIHC, Liver hepatocellular carcinoma; LUAD, Lung adenocarcinoma; LUSC, Lung squamous cell carcinoma; PAAD, Pancreatic adenocarcinoma; PRAD, Prostate adenocarcinoma; PCPG, Pheochromocytoma and Paraganglioma; READ, Rectum adenocarcinoma; SARC, Sarcoma; SKCM, Skin Cutaneous Melanoma; LAML, Acute Myeloid Leukemia; TGCT, Testicular Germ Cell Tumors; THCA, Thyroid carcinoma; THYM, Thymoma; STAD, Stomach Adenocarcinoma; UCEC, Uterine Corpus Endometrial Carcinoma; UCS, Uterine Carcinosarcoma; UVM, Uveal Melanoma

Supplementary Figure S3:

Kaplan-Meier survival curves for 47 patients with DLBCL who were included in the TCGA database, stratified by the Siglec-15 mRNA expression level (high or low/medium). High expression was defined as an expression value that was higher than the 3^rd^ quartile.
