# Supplementary figures and images for: Sialic acid-binding immunoglobulin-like lectin-15 expression on peritumoral macrophages is a favorable prognostic factor for primary central nervous system lymphoma patients
Source: Sci Rep. 2021 Jan 13;11:1206. doi: 10.1038/s41598-020-79742-9 (PMC7806611; doi:10.1038/s41598-020-79742-9)

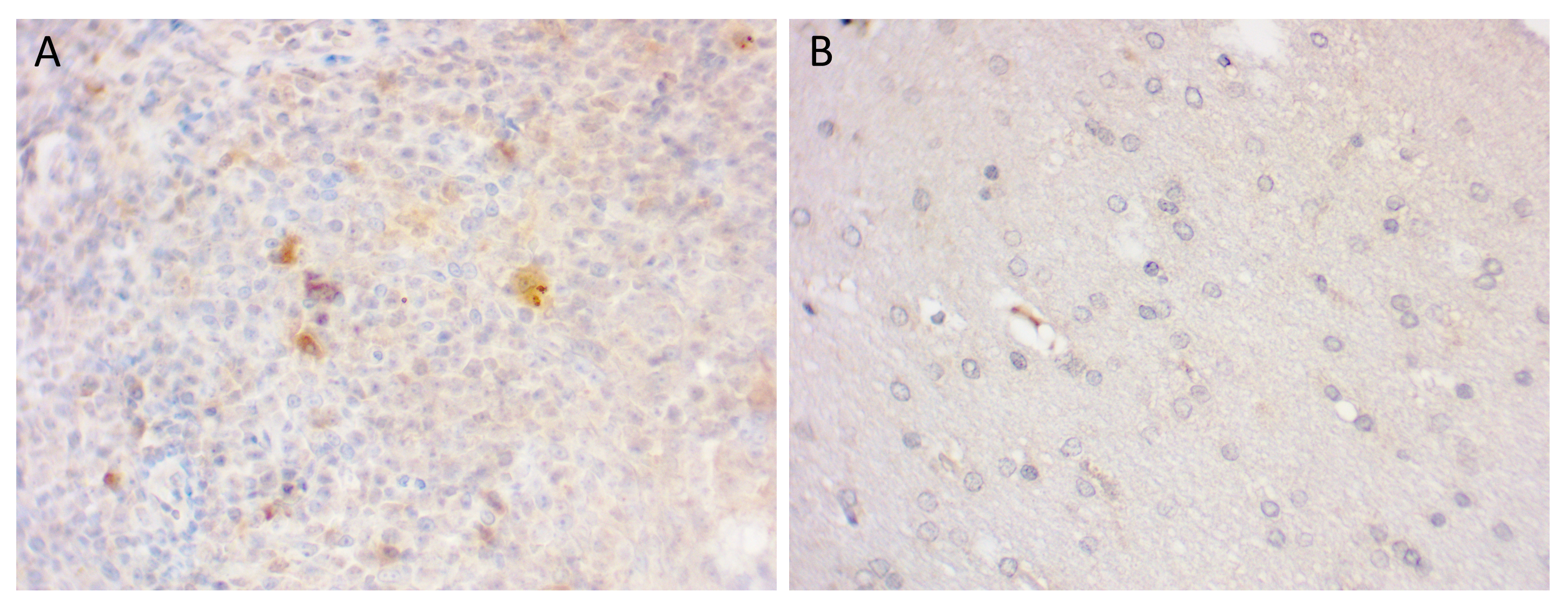

Supplement: Supplementary file 2 — Supplementary Figure S1. [file 41598_2020_79742_MOESM2_ESM.tif]

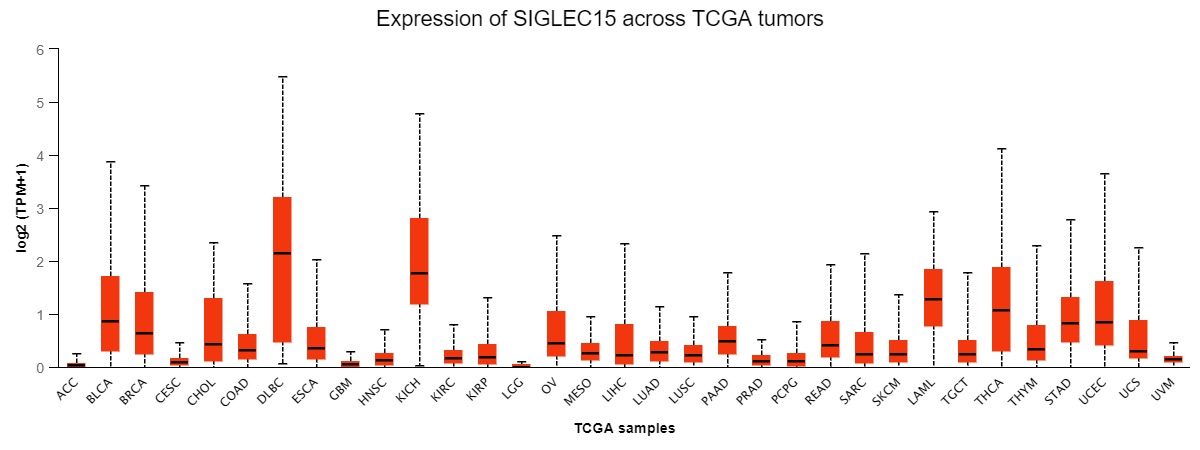

Supplement: Supplementary file 3 — Supplementary Figure S2. [file 41598_2020_79742_MOESM3_ESM.jpeg]
